# Efficacy of Continuous S(+)-Ketamine Infusion for Postoperative Pain Control: A Randomized Placebo-Controlled Trial

**DOI:** 10.1155/2016/6918327

**Published:** 2016-02-02

**Authors:** Luiz Eduardo de Paula Gomes Miziara, Ricardo Francisco Simoni, Luís Otávio Esteves, Luis Henrique Cangiani, Gil Fernando Ribeiro Grillo-Filho, Anderson Garcia Lima e Paula

**Affiliations:** ^1^Department of Anesthesiology, Centro Médico Campinas, Rua Edilberto Luis Pereira da Silva 150, Campinas, SP, Brazil; ^2^Department of Pharmacology, Universidade de Campinas (UNICAMP), Centro Médico Campinas, Rua Edilberto Luis Pereira da Silva 150, 13083-190 Campinas, SP, Brazil

## Abstract

*Aim.* A double-blind, randomized, placebo-controlled trial was designed to evaluate the efficacy of continuous intraoperative infusion of S(+)-ketamine under intravenous anesthesia with target-controlled infusion of remifentanil and propofol for postoperative pain control.* Methods.* Forty-eight patients undergoing laparoscopic cholecystectomy were assigned to receive continuous S(+)-ketamine infusion at a rate of 0.3 mg·kg^−1^·h^−1^ (*n* = 24, intervention group) or an equivalent volume of saline at the same rate (*n* = 24, placebo group). The same target-controlled intravenous anesthesia was induced in both groups. Pain was assessed using a 0 to 10 verbal numeric rating scale during the first 12 postoperative hours. Pain scores and morphine consumption were recorded in the postanesthesia care unit (PACU) and at 4 and 12 hours after surgery.* Results.* Pain scores were lower in the intervention group at all time points. Morphine consumption did not differ significantly between groups during PACU stay, but it was significantly lower in the intervention group at each time point after PACU discharge (*P* = 0.0061). At 12 hours after surgery, cumulative morphine consumption was also lower in the intervention group (5.200 ± 2.707) than in the placebo group (7.525 ± 1.872).* Conclusions.* Continuous S(+)-ketamine infusion during laparoscopic cholecystectomy under target-controlled intravenous anesthesia provided better postoperative pain control than placebo, reducing morphine requirement.* Trial Registration*. This trial is registered with ClinicalTrials.gov NCT02421913.

## 1. Introduction

Postoperative pain control with multimodal analgesia is important not only to relieve pain but also to reduce postoperative side effects, such as nausea, vomiting, need for sedation, and length of hospital stay. Therefore, adjuvant drugs with proven efficacy in this type of analgesia are very useful for anesthesiologists.

Several experimental and clinical studies suggest that the administration of potent short-acting opioids, such as remifentanil, is associated with central sensitization to pain, a phenomenon known as opioid-induced hyperalgesia [1–4]. The activation of N-methyl-D-aspartate (NMDA) receptors has been suggested to be the main mechanism facilitating the response to sensory stimuli and leading to opioid-induced hyperalgesia, along with a state of excitability in the spinal cord dorsal horn.

One of the two subunits of the NMDA, namely, subunit 2B (NR2B), has an important role in the genesis of remifentanil-induced hyperalgesia and inflammatory hyperalgesia caused by the induction of long-term potentiation. A previous study found that increased tyrosine phosphorylation in this subunit may be prevented with the use of ketamine [5].

Although ketamine acts on several receptors, its analgesic effects result mainly from the fact that it is a NMDA receptor antagonist that prevents central sensitization [2, 6] and inhibits this receptor by reducing mean time and frequency of NMDA receptor channel opening, the latter through an allosteric mechanism. Therefore, ketamine has been widely used to investigate the role of the NMDA receptor in several animal and human models.

S(+)-ketamine is a ketamine isomer that acts by changing channel opening time through noncompetitive blockade [7]. Furthermore, compared with the racemic mixture, it has a twofold higher analgesic potency [7, 8] and a three- to fourfold greater affinity for the intrachannel phencyclidine receptor site, which is the site of action of S(+)-ketamine.

The present study was designed to evaluate the efficacy of continuous intraoperative infusion of S(+)-ketamine for pain control after laparoscopic cholecystectomy under target-controlled total intravenous anesthesia, testing the hypothesis that S(+)-ketamine is more effective than placebo in the control of postoperative pain.

## 2. Methods

This double-blind, randomized, placebo-controlled trial was conducted at Centro Médico Campinas, Brazil, from June 2012 to February 2014, and participants were recruited among patients scheduled for laparoscopic cholecystectomy during this period. After approval of the study by the Research Ethics Committee, all participants signed the informed consent form. The trial is registered at ClinicalTrials.gov, number NCT02421913. Eligible participants were all patients aged 18–65 years with American Society of Anesthesiologists Physical Status (ASA PS) classes 1-2. Exclusion criteria were use of alcohol or illicit drugs, H2 inhibitors, opioids, or calcium channel blockers in the last 10 days, chronic pain, myocardial ischemia, or psychiatric disorders.

Sample size was calculated based on the results of a previous pilot study. To detect a difference of at least 38% in pain scores in the postanesthesia care unit (PACU) between the intervention and placebo groups, with an alpha error of 5% and a beta error of 20%, a sample size of at least 21 patients per group was required to achieve 80% power and a 95% confidence interval. Three more patients were added to each group to account for possible losses to follow-up during the trial.

Participants were randomly assigned in a 1 : 1 ratio to receive either S(+)-ketamine or placebo during laparoscopic cholecystectomy. The randomization sequence was created by an anesthesiologist with no involvement in the care of participants using the Randomizer software (https://www.randomizer.org/). All participants and the anesthesiologist performing the laparoscopic cholecystectomy were kept blind to group assigment. The allocation sequence was concealed in sequentially numbered, sealed envelopes. In order to preserve the double-blind nature of the study, the envelopes were opened by a third person with no involvement in the anesthetic procedure, who prepared a 50 mL syringe containing either S(+)-ketamine (intervention group) or saline (placebo group). In case of emergency, the anesthesiologist responsible for the care of the patient was allowed to violate the protocol and be informed about the group to which the patient was assigned.

No preanesthetic medication was administered to patients. Mean noninvasive blood pressure (MBP), ECG, pulse oximetry (SpO_2_), bispectral index (BIS), and capnography were monitored in all patients after tracheal intubation.

After venipuncture, patients received intravenous parecoxib sodium (40 mg). Anesthesia was induced with midazolam at a dose of 0.05 mg·kg^−1^ and target-controlled infusions of propofol (target dose of 3.0 *μ*·mL^−1^) and remifentanil (target dose of 6.0 ng·mL^−1^) using the Marsh pharmacokinetic model with ke0 of 1.21 min^−1^ and the Minto pharmacokinetic model, respectively. Unconsciousness was determined by loss of corneal and palpebral reflexes and confirmed by a BIS < 50. Rocuronium (0.6 mg·kg^−1^) was then administered. Immediately after tracheal intubation, the target dose of propofol was adjusted to maintain BIS between 35 and 50 and the target dose of remifentanil was reduced to 3 ng·mL^−1^. Five minutes before surgery, patients assigned to the intervention group received continuous S(+)-ketamine infusion at a rate of 0.3 mg·kg^−1^·h^−1^, and patients assigned to the placebo group received an equivalent volume of saline at the same rate.

At the beginning of surgery, the target dose of remifentanil was increased to 5 ng·mL and adjusted intraoperatively to maintain MBP ± 15% of baseline levels, while the target dose of propofol was adjusted intraoperatively to maintain BIS between 35 and 50. At the end of the procedure, neuromuscular blockade was reversed with sugammadex. Additionally, propofol and remifentanil infusions were discontinued, as well as S(+)-ketamine and saline infusions in the intervention and placebo groups.

The duration of anesthesia, duration of surgery, time until awakening (spontaneous opening of the eyes and/or BIS > 70), and overall dose of S(+)-ketamine, remifentanil, and propofol infused per total body weight were recorded. Patients were extubated in the operating room and transferred to the PACU, where postoperative pain was assessed using a 0 to 10 verbal numeric scale (VNS). Patients were considered pain-free if they scored ≤2 and in pain if they scored ≥3 on the VNS. Morphine was administered at a dose of 0.05 mg·kg^−1^ when the patient reported pain for the first time and at a dose of 0.025 mg·kg^−1^ on subsequent occasions. Pain scores were recorded in the PACU and at 4 and 12 hours after the end of surgery, and patients were continuously monitored during their PACU stay by one of the anesthesiologists responsible for this clinical trial. Patients were assessed by the same anesthesiologist every 20 minutes after being discharged from PACU and within the first 4 hours after surgery and every 40 minutes from the fourth to the twelfth hour after surgery. All patients were instructed to call for the presence of the anesthesiologist responsible for the surgical procedure, either verbally or using an individual alarm device, at any time between these assessments in case of pain or any other unpleasant effect.

Parametric variables were analyzed using Student's *t*-test, and nonparametric variables were analyzed using the Mann-Whitney *U* test. Variables were expressed as mean and standard deviation (SD). Differences were considered significant when the *P* value was less than 0.05.

## 3. Results

**Figure 1:**
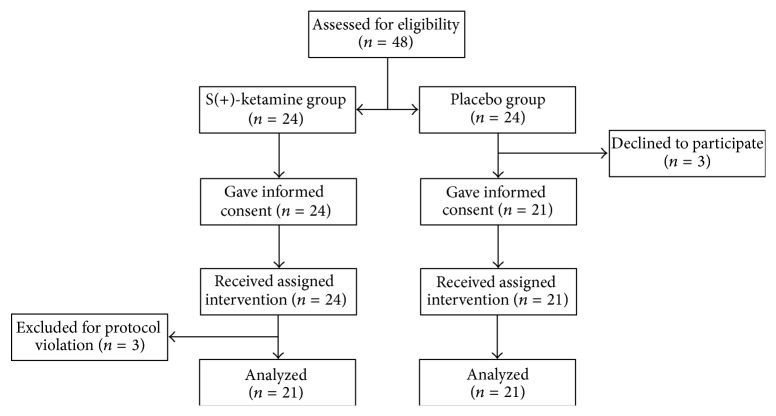
Patient flow chart.

A total of 48 patients were enrolled in the trial: 24 were randomized to S(+)-ketamine and 24 to placebo. Three patients from the placebo group refused to participate in the study. Of 24 patients who received continuous S(+)-ketamine infusion, three were excluded for protocol violation. Therefore, the final analysis included 42 patients, 21 in the S(+)-ketamine group and 21 in the placebo group (Figure 1).

Patients' demographic data were similar in both groups. There was no difference between groups regarding duration of surgery, duration of anesthesia, and time until awakening. There was no significant statistical difference between the two groups with regard to length of stay, but all patients stayed at the unit for 120 minutes for a more accurate evaluation of the outcomes of interest.

Mean (SD) remifentanil consumption was 0.170 (0.054) mcg·kg^−1^·min^−1^ in the S(+)-ketamine group and 0.228 (0.042) mcg·kg^−1^·min^−1^ (*P* = 0.0175) in the placebo group, and mean (SD) propofol consumption was 72.194 (11.539) mcg·kg^−1^·min^−1^ in the S(+)-ketamine group and 84.895 (13.739) mcg·kg^−1^·min^−1^ in the placebo group, with a significant difference between groups (*P* = 0.0286).

**Figure 2:**
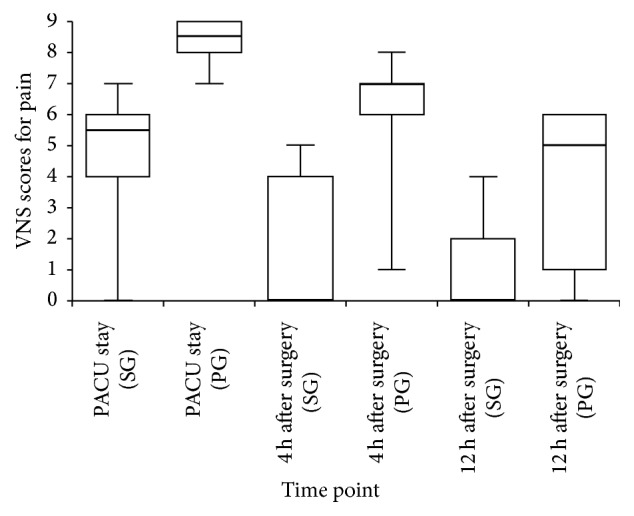
Pain scores during PACU stay and at 4 and 12 h after surgery. PACU: postanesthesia care unit; PG: placebo group; SG: S(+)-ketamine group.

Median pain scores on the VNS during PACU stay were 5.5 in the S(+)-ketamine group and 8.5 in the placebo group (*P* < 0.0001). Pain scores decreased to 0.0 in the S(+)-ketamine group and 7.0 in the placebo group (*P* = 0.0004) at 4 hours after surgery and to 0.0 and 5.0, respectively, at 12 hours after surgery (*P* = 0.0309) (Figure 2).

**Figure 3:**
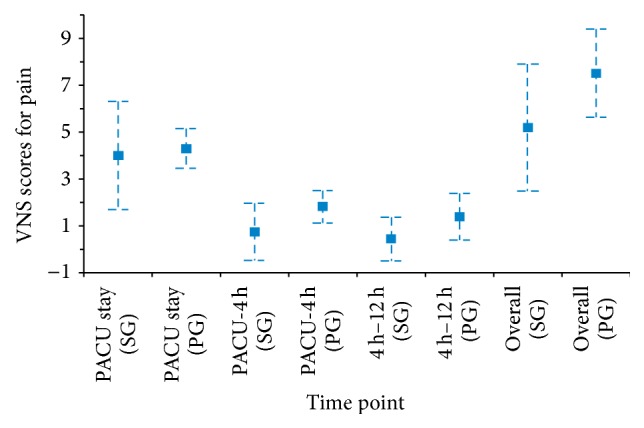
Morphine consumption during PACU stay and at 4 and 12 h after surgery. 4 h–12 h: from 4 to 12 h after surgery; PACU: postanesthesia care unit; PACU-4 h: from PACU discharge up to 4 h after surgery; PG: placebo group; SG: S(+)-ketamine group.

There was no difference in morphine consumption during PACU stay between patients who received S(+)-ketamine (4.00 [SD, 2.29] mg) and placebo (4.30 [SD, 0.83] mg) (*P* = 0.5770). The mean (SD) dose of morphine used was 0.750 (1.198) mg in the S(+)-ketamine group and 1.825 (0.689) mg in the placebo group (*P* = 0.0108) from PACU discharge up to 4 hours after surgery and 0.450 (0.93) mg and 1.400 (0.99) mg, respectively, (*P* = 0.0089) between 4 and 12 hours after surgery, with statistically significant differences at these time points. Cumulative morphine consumption was significantly lower in the S(+)-ketamine group than in the placebo group (5.200 [SD, 2.707] mg versus 7.525 [SD, 1.872] mg; *P* = 0.0061) (Figure 3).

The following postoperative side effects were recorded: nausea (1 patient in the S(+)-ketamine and 2 patients in the placebo group), agitation (2 patients in the S(+)-ketamine group), and hallucination (1 patient in the S(+)-ketamine group), with no difference between groups.

## 4. Discussion

In the current study, continuous intraoperative infusion of S(+)-ketamine (at a rate of 0.3 mg·kg^−1^·h^−1^) in patients under intravenous anesthesia with target-controlled infusion of remifentanil and propofol provided better postoperative pain control than placebo over the first 12 hours after laparoscopic cholecystectomy.

Continuous infusion of remifentanil has been associated with the development of opioid-induced hyperalgesia [9, 10]. Experimental studies have shown that patients become rapidly tolerant to infusion of potent short-acting opioids such as remifentanil. The mechanisms involved in this tolerance include activation of NMDA receptors in the spinal cord dorsal horn [2, 6, 11], inactivation of *μ*-opioid receptors [12], spinal dynorphin release [13], and upregulation of cyclic adenosine monophosphate [14].

The time of recovery from anesthesia with remifentanil is relatively dose independent. Thus, this drug can be administered at high doses during surgery with low risk of delayed postoperative recovery or respiratory depression. However, high doses of remifentanil administered intraoperatively are more closely related to the induction of postoperative secondary hyperalgesia as compared to long-acting opioids [2, 4, 15], requiring higher doses of morphine in the postoperative period to provide appropriate analgesia [16]. In a study with healthy human volunteers, a skin area with preexisting mechanical hyperalgesia was significantly enlarged 60–90 minutes after remifentanil infusion [17].

NMDA receptors are complexes formed by at least two types of subunits: NR1A/B and NR1A/2B. Subunit 2B (NR2B) is particularly important, because of its role in the organization of sensory pathways and in the development of neuropathic pain [18, 19].

A previous study has shown that propofol acts as a NMDA receptor antagonist on the NR1 subunit. This phenomenon was mediated by a signaling mechanism involving the activation of protein phosphatase 2A and thus may have some effect on reducing opioid-induced hyperalgesia [20].

Tyrosine phosphorylation in the NR2B subunit has an important role in the induction of long-term potentiation, a phenomenon associated with central sensitization [21] and with the onset and development of inflammatory hyperalgesia [11]. Remifentanil-induced hyperalgesia has been shown to lead to a considerable increase in tyrosine phosphorylation in the NR2B subunit, and this increase may be prevented by ketamine [5]. Yuan et al. suggest that glycogen synthase kinase- (GSK-) 3 beta contributes to the development of remifentanil-induced hyperalgesia through the regulation of NMDA receptor subunits (NR1 and NR2B) in the spinal cord and that inhibition of GSK-3 beta may be effective in the treatment of hyperalgesia [22].

Kingston et al. [20] showed the antagonist effect of propofol on NMDA receptors. Cerebral mechanisms may be important for the results obtained by the interaction between remifentanil and S-ketamine, because several pain pathways are influenced differently by one of the two drugs. Remifentanil directly inhibits nociceptive information through opioid receptors located in the nervous system, whereas S(+)-ketamine acts on brain structures such as insula and anterior cortex, thus modulating the emotional aspect of pain [23]. Gupta et al. suggest an important synergism between ketamine and opioids [24]. In the S(+)-ketamine group, there was a decrease in propofol and remifentanil consumption, but future studies are needed to establish more accurate correlations and conclusions on the specific interactions between these drugs.

The blockade of NMDA receptor with the administration of ketamine suppressed the development of opioid-induced hyperalgesia and injury-induced central sensitization even when ketamine was given at subanesthetic doses [25]. Clinical studies have demonstrated the efficacy of ketamine in the control of acute postoperative pain in several surgical procedures. Results have shown reduced pain scores and analgesic consumption even after the end of the duration of action of ketamine.

A systematic review concluded that intravenous ketamine is an effective adjunct for postoperative analgesia, being particularly beneficial in abdominal, thoracic, and orthopedic surgeries [6]. However, an opposite result was found by a meta-analysis on the use of NMDA receptor antagonists to control pain and remifentanil-induced hyperalgesia [2].

The reduced remifentanil consumption in the S(+)-ketamine group may have contributed to reduction of post-operative hyperalgesia. Guignard et al. concluded that higher doses of remifentanil in the intraoperative period lead to an increase in pain and in the postoperative consumption of morphine [9].

Hang et al. concluded that the effective dose of ketamine in 50% and 95% of patients (ED_50_ and ED_95_) for prevention of postoperative hyperalgesia after remifentanil-based anesthesia was 0.24 mg/kg and 0.33 mg/kg, respectively [15]. In another study, Untergehrer et al. used S(+)-ketamine at continuous infusion rates from 0.25 to 0.50 mg·kg·h as subanesthetic doses [1].

The rate of continuous S(+)-ketamine infusion used in this study (0.3 mg·kg^−1^·h^−1^) is associated with effective analgesia [1, 15], whereas higher doses of S(+)-ketamine are associated with side effects leading to cognitive changes, such as hallucinations and unpleasant dreams, and with mood, perception, and consciousness changes. According to the pharmacokinetic model proposed by White et al. [26], a 0.3 mg/kg/h infusion for 16 minutes would lead to a concentration effect of 70 ng/mL. Since our surgical team started to perform surgery 11 minutes after anesthetic induction, on average, we decided to start the infusion 5 minutes before anesthetic induction. Koppert et al. used a similar dose of S ketamine in another study, also without an initial bolus [27].

The current study evaluated the effect of S(+)-ketamine using pain scores and overall consumption of morphine recorded over a 12-hour period after the end of surgery. The presence of any postoperative side effects was also noted. Despite the lower morphine requirement in the S(+)-ketamine group, the incidence of postoperative side effects did not differ significantly between groups.

The main limitation of this study is the lack of a longer follow-up throughout the postoperative period, which prevented us from drawing a more comprehensive conclusion on postoperative pain control. The decision to evaluate patients throughout a 12-hour period is based on the mean postoperative length of stay of patients undergoing laparoscopic cholecystectomy in our service. In addition, we believe that studying pain control in this surgery is of paramount importance, because this procedure is capable of releasing nociceptive mediators and requires a short hospital stay [28].

## 5. Conclusions

The present study tested the hypothesis that continuous infusion of low-dose S(+)-ketamine is more effective than placebo in the control of postoperative pain. Despite the lack of statistical difference in morphine consumption during PACU stay, this hypothesis is supported by our findings that continuous S(+)-ketamine infusion at a rate of 0.3 mg·kg^−1^·h^−1^ during laparoscopic cholecystectomy under target-controlled intravenous anesthesia reduced the overall need for postoperative morphine and VNS pain scores at all time points assessed. However, we recognize that further studies with longer postoperative follow-up are required to better understand the correlations found in this study.

## Abbreviations

### Abbreviations

ASA PS: American Society of Anesthesiologists Physical Status
GSK: Glycogen synthase kinase
NMDA: N-Methyl-D-aspartate
PACU: Postanesthesia care unit
VNS: Verbal numeric scale.
